# In the aftermath of clozapine discontinuation: comparative effectiveness and safety of antipsychotics in patients with schizophrenia who discontinue clozapine

**DOI:** 10.1192/bjp.2019.267

**Published:** 2020-09

**Authors:** Jurjen J. Luykx, Noraly Stam, Antti Tanskanen, Jari Tiihonen, Heidi Taipale

**Affiliations:** 1 Assistant Professor, Departmentsof Psychiatry and Translational Neuroscience, UMC Utrecht Brain Center, University Medical Center Utrecht, Utrecht University; and Department of Outpatient Psychiatry, GGNet Mental Health, The Netherlands; 2 Researcher, Department of Psychiatry, UMC Utrecht Brain Center, University Medical Center Utrecht, Utrecht University, The Netherlands; 3 Researcher, Department of Clinical Neuroscience, Karolinska Institutet, Sweden; Department of Forensic Psychiatry, University of Eastern Finland, Niuvanniemi Hospital; and Public Health Solutions, National Institute for Health and Welfare, Finland; 4 Professor, Department of Clinical Neuroscience, Karolinska Institutet, Sweden; Department of Forensic Psychiatry, University of Eastern Finland, Niuvanniemi Hospital, Finland; and Center for Psychiatric Research, Stockholm City Council, Sweden; 5 Assistant Professor, Department of Clinical Neuroscience, Karolinska Institutet, Sweden; Academy Research Fellow, Department of Forensic Psychiatry, University of Eastern Finland, Niuvanniemi Hospital; and Associate Professor, School of Pharmacy, University of Eastern Finland, Finland

**Keywords:** Schizophrenia, antipsychotics, clozapine, psychiatric ward readmission, discontinuation

## Abstract

**Background:**

Although clozapine is often discontinued, there is a paucity of guidelines and evidence on treatment options after clozapine discontinuation. Moreover, it is currently unknown whether reinstating clozapine in patients formerly using clozapine should be avoided.

**Aims:**

To compare the real-world effectiveness of antipsychotics after clozapine cessation.

**Method:**

From Finnish registry data (1995–2017), we identified 2250 patients with schizophrenia who had been using clozapine for ≥1 year before treatment cessation. The primary analysis consisted of adjusted within-individual analyses of psychiatric ward readmission owing to psychosis and treatment failure. Secondary analyses concerned between-individual mortality differences.

**Results:**

Compared with no use of antipsychotics, risk of psychiatric ward readmission was lowest for reinitiation of clozapine (adjusted hazard ratio (aHR) 0.49; 95% CI 0.40–0.61; *P* < 0.0001), oral olanzapine (aHR 0.58; 95% CI 0.48–0.71; *P* < 0.0001) and antipsychotic polypharmacy (aHR 0.62; 95% CI 0.53–0.72; *P* < 0.0001). Risk of treatment failure was lowest for aripiprazole long acting injectable (aHR 0.42; 95% CI 0.27–0.65; *P* < 0.0001), reinitiation of clozapine (aHR 0.49; 95% CI 0.43–0.57; *P* < 0.0001) and oral olanzapine (aHR 0.69; 95% CI 0.61–0.77; *P* < 0.0001). Mortality risk was lowest for reinitiation of clozapine (aHR 0.18; 95% CI 0.09–0.36; *P* < 0.0001) and oral olanzapine (aHR 0.26; 95% CI 0.17–0.40; *P* < 0.0001).

**Conclusions:**

Clozapine and olanzapine are the most effective and safest treatment options in those discontinuing clozapine for undefined reasons. Clozapine should therefore be reconsidered in patients with schizophrenia who previously discontinued this compound.

## Current treatment options after clozapine cessation

Clozapine has proven superior efficacy over other antipsychotics^1,2^ and is also the only drug registered for treatment of therapy-resistant schizophrenia (TRS) in several countries.^2,3^ Clozapine is superior to other antipsychotics in reducing positive symptoms and psychiatric ward admissions in patients with TRS.^4,5^ TRS is commonly defined as ‘failure to respond to two or more antipsychotics (one of which should be a second-generation antipsychotic) given in an adequate dose for at least 6–8 weeks’.^6,7^ Around 30% of patients with schizophrenia are treatment resistant.^8,9^ Of those, approximately 60% do not respond to clozapine.^10^ Moreover, around 20% of those who initiate clozapine discontinue treatment because of limited tolerability.^11,12^

For those unresponsive to clozapine, three strategies may be tried: augmentation with non-antipsychotic treatment modalities, combinations of antipsychotics and switching. At present, guidelines do not provide a clear answer as to the preferred of those options for patients who discontinue their treatment with clozapine.^6,8^ On a similar note, a recent meta-analysis on clozapine combination and augmentation strategies in patients with TRS concluded that most studies supporting such strategies are of low quality.^13^ Moreover, when only high-quality studies with sufficient numbers of participants are included in subanalyses, little benefit is found for pharmacological augmentation and combination strategies, with the exception of electroconvulsive therapy being supported in one high-quality study.^13^ Furthermore, to our knowledge no randomised trials into treatment options in patients with schizophrenia who are intolerant or unresponsive to clozapine have been published. In sum, possibly because of a lack of power and the current paucity of high-quality clinical trials, little is known about the optimal treatment strategies in those discontinuing clozapine.

## Real-world studies versus randomised trials

A statistically powerful approach to shed light on the comparative effectiveness of treatment options in schizophrenia is a large and unselected registry study. Randomised clinical trials (RCTs) often focus on short-term symptom reduction. Because schizophrenia is a lifelong illness, observational studies could reflect real-world impact better than RCTs by focusing on long-term outcomes, such as relapse prevention.^14,15^ By ‘real world’, we refer to data drawn from registry and observational studies. Furthermore, patients who enrol in RCTs often have better treatment adherence and less severe symptoms than patients in a naturalistic setting, hampering generalisability to clinical practice.^16^ To minimise this issue of selection bias (i.e. selective inclusion of participants leading to spurious findings), a potential pitfall in observational studies, within-individual analyses may be applied to national registry data. Exposure periods of each individual may thus be compared with non-exposure periods of the same individual, a strategy with proven benefit to resolve important clinical dilemmas.^14,15^

## Rationale and aims of the current study

Given the lack of guidelines and evidence on treatment options after clozapine discontinuation, we set out to investigate the comparative effectiveness (i.e. treatment benefit ascertained in a non-clinical trial setting) of pharmacological treatment options in patients with schizophrenia who terminate clozapine treatment. We hypothesised that in patients formerly using clozapine, this compound should be best avoided because of either limited efficacy or lack of tolerability, reasoning that patients who had discontinued clozapine would be relatively resistant to its efficacy and/or susceptible to its adverse drug reactions. Moreover, there is also evidence of reduced effectiveness of antipsychotics after reintroduction or sustained use, in particular for clozapine.^17–19^ We realise that discontinuing clozapine may follow more than one pathway, primarily lack of efficacy, low tolerability and patients’ decisions. Importantly, such scenarios reflect clinical practice where patients may stop clozapine for unknown reasons or stop attending follow-up consultations. Moreover, physicians are often unable to retrospectively assess the reason for clozapine discontinuation in a given patient. Given the estimated fairly high likelihood of non-responsiveness to clozapine in patients with TRS (60%),^10^ we conducted a national registry study applying within- and between-individual analyses to a range of clinically relevant outcomes in patients with schizophrenia spectrum disorder who discontinue clozapine. We thus explored which real-world antipsychotic treatment performs best in those previously taking clozapine.

## Method

This project was approved by the Ethics Committee of the Finnish National Institute for Health and Welfare (dated 4 December 2013; 8/2013). Further permissions were granted by pertinent institutional authorities at the Finnish National Institute for Health and Welfare (permission THL/1466/6.02.00/2013), the Social Insurance Institution of Finland (34/522/2013) and Statistics Finland (TK53-305-13). We abided by the declaration of Helsinki. According to Finnish legislation, informed consent is not required for register-based studies using pseudonymised data.

We first identified all persons diagnosed with schizophrenia spectrum disorders (ICD-10 codes F20 and F25,^20^ and ICD-9^21^ and ICD-8 code 295*^22^) in Finland from 1972 to 2014 (*N* = 62 250).^23^ These data were extracted from the Hospital Discharge Register maintained by the Finnish National Institute of Health and Welfare, based on in-patient hospital care diagnoses. From the Hospital Discharge Register (https://thl.fi/en/web/thlfi-en/statistics/information-on-statistics/register-descriptions/care-register-for-health-care), all hospital care periods with corresponding discharge diagnoses from 1972 until 2017 were identified. Data was also collected from the Prescription Register (https://www.kela.fi/web/en/492; 1995–2017, maintained by the Social Insurance Institution) and dates of death from the Social Insurance Institution database (1972–2017) were linked with the Population Register System. The Prescription Register data included all reimbursed drug dispensings from Finnish pharmacies, with information on purchased amount (in defined daily doses), dispensed drug and drug substance coded with Anatomical Therapeutic Chemical (ATC) codes (http://www.whocc.no/atc/structure_and_principles/). All residents of Finland have been assigned a unique personal identification number, which enables linkage between nationwide registers.

Antipsychotic use was identified with ATC code N05A, with the exception of lithium (which is listed under N05A). Antipsychotics were categorised into oral and long-acting injectable antipsychotics (LAIs) according to the drug product information in the Prescription Register data. Drug use periods were constructed from dispensings by the PRE2DUP method.^24^ The method is based on modelling of each drug (per drug form) for each person separately, by computing sliding averages of defined daily doses according to individual drug use patterns. The PRE2DUP method takes into account ward admissions (when drugs are provided by the caring unit and not recorded in the registers), stockpiling of drugs and changes in doses.

For the current analyses, we identified all clozapine use periods that ended with discontinuation of use (*n* = 7037). To ensure that patients had been stable on clozapine before discontinuation, we required that clozapine use had been ongoing for at least 1 year (*n* = 3585 use periods). We excluded clozapine use periods that were followed by clozapine reinitiation within <1 year. From the remaining 2313 use periods, we chose the first use period per person, resulting in the final study population of 2250 clozapine discontinuers (Supplementary Fig. 1 available at https://doi.org/10.1192/bjp.2019.267).

The main outcome variables in our study used for our primary analyses were psychiatric ward readmission (psychosis determined as ICD-10 codes F20–F29 recorded as the main diagnosis of admission) and treatment failure, which encompassed (a) any changes in antipsychotic treatment (switching, addition of another antipsychotic compound and/or stopping the use of the current antipsychotic), (b) any psychiatric disorder–related admission, such as substance misuse or depressive episodes (ICD-10 codes F00–F99) and (c) death. We thus regard these two primary outcomes measures to index effectiveness. Secondary analyses were conducted with all-cause mortality as an outcome event. Here, we thus regard mortality as a proxy for safety, meaning that where we refer to safety in the manuscript, we derive this from mortality figures. Causes of death were categorised as unnatural (ICD-10 codes V01–Y98) or natural/undetermined (the rest). In addition, we assessed the proportion of clozapine discontinuers who had received diagnoses of neutropenia or agranulocytosis (ICD-10 code D70), myocarditis (ICD-10 codes I40, I41, I51.4 and I09.0) and ileus (ICD-10 codes K56 and K31.5) during the 3 months preceding clozapine discontinuation in in-patient or specialised out-patient care visits.

The follow-up in this study started at discontinuation of clozapine and ended with either death or end of study follow-up, i.e. 31 December 2017. As some persons re-started clozapine during follow-up, sensitivity analyses were conducted by excluding those who reinitiated clozapine. Our primary analyses of psychiatric ward readmissions and treatment failure were performed with a within-individual design and these events were treated as recurrent events. Within-individual results were analysed with stratified Cox proportional hazard regression models in which each individual formed his or her own stratum.^25^ In within-individual analyses, the follow-up time for each individual was reset to zero after each outcome event, and all time-invariant covariates were controlled for in the design. Only persons having an outcome event contributed to within-individual analyses. Within-individual analyses were adjusted for time-varying covariates, i.e. sequential order of treatments, use of antidepressants (ATC code N06A), benzodiazepines and related drugs (ATC codes N05BA, N05CD and N05CF), mood stabilisers (ATC codes: valproate N03AG01, carbamazepine N03AF01, lamotrigine N03AX09 and lithium N05AN01) and time since cohort entry (i.e. the moment of clozapine discontinuation).

Additional sensitivity analyses were conducted with between-individual, traditional, multivariate-adjusted Cox regression models. These analyses were adjusted for gender, age at clozapine discontinuation, the number of previous ward admissions owing to psychosis, time since first schizophrenia diagnosis and, in addition, continuously updated variables for current versus no use of medications (lipid-modifying agents, opioid analgesics, non-opioid analgesics, anti-Parkinson drugs and prior use of LAI) and continuously updated variables for the following diagnoses: substance misuse, cardiovascular disease, diabetes, asthma/chronic obstructive pulmonary disease, previous cancer, renal disease or previous suicide attempt. Definitions of comorbidities and other medications are described in Supplementary Table 1. Only out-patient care deaths (≤2 days in ward care) were included in mortality analyses because drug use during ward stays is not recorded in the register data.

All periods including more than one antipsychotic were coded as ‘antipsychotic polypharmacy’.^14^ Time was assigned to each exposure. We present the results of antipsychotics with ≥10 events for the main outcomes (oral antipsychotics if not otherwise stated): chlorpromazine, levomepromazine, perphenazine, perphenazine LAI, haloperidol, zuclopenthixol LAI, clozapine, olanzapine, olanzapine LAI, quetiapine, risperidone, risperidone LAI, aripiprazole and aripiprazole LAI; all other antipsychotics in monotherapy are one category (‘other antipsychotics’). For mortality analyses we applied between-individual, multivariate-adjusted Cox regression of only those agents with four or more events: levomepromazine, clozapine, olanzapine, quetiapine, risperidone and aripiprazole. Separate analyses were conducted to investigate specific combinations of antipsychotics. In these analyses, psychiatric ward admission and treatment failure outcomes were analysed by considering two-drug combinations of the five most commonly used oral antipsychotics in the study population: clozapine, olanzapine, quetiapine, risperidone and aripiprazole.

The results are presented as adjusted hazard ratios (aHRs) with 95% confidence intervals, adjusted for the abovementioned covariates. The level of statistical significance was set at *P* < 0.003125 after Bonferroni correction for 16 agents studied (0.05/16 = 0.003125), and the results that were significant after this correction are shown in bold in figures; 95% confidence intervals are also provided. The authors had full and ongoing access to the study data, which are available upon reasonable request.

## Results

A total of 7037 patients received clozapine monotherapy and of these, 49.1% (*n* = 3452) discontinued therapy within 1 year. Of those 3452 individuals, 2250 met our inclusion criteria. In this final study population, participants were somewhat more likely to be male (57%) than female, with a median age of 46 years (Table 1). They had been using clozapine for a median of 4.1 (interquartile range, 2.0–8.0) years before discontinuation. A total of 42% (*n* = 934) had been admitted to a ward during a period of 3 months before clozapine cessation. Of these, the majority (55.5%, *n* = 517) were for psychiatric reasons (mainly schizophrenia, *n* = 482), followed by diseases of the respiratory system (9.8%, *n* = 91, of which pneumonia was the most common reason).

**Table 1:** Descriptive statistics of all clozapine discontinuers, and those who re-started versus those who did not re-start clozapine during follow-up

	All clozapine discontinuers, *N* = 2250	Those who did not re-start clozapine during the follow-up, *n* = 1871	Those who re-started clozapine during the follow-up but ≥1 year after clozapine cessation, *n* = 379	*P*-value^a^
Male gender, percentage (number)	57.2 (1287)	56.4 (1056)	61.0 (231)	0.1057
Median age at schizophrenia diagnoses (IQR), years	27 (22–35)	28 (23–36)	25 (22–31)	<0.0001
Median age at clozapine discontinuation (IQR), years	46 (34–58)	48 (36–59)	38 (30–48)	<0.0001
Median duration of clozapine use before discontinuation (IQR), years	4.1 (2.0–8.0)	4.5 (2.2–8.5)	2.9 (1.7–5.8)	<0.0001
Median time to clozapine reinitiation (IQR), years			2.1 (1.4–4.3)	
Most frequently initiated first antipsychotics during the first year after clozapine discontinuation^b^			
Antipsychotic polypharmacy	26.5 (409)	27.0 (350)	24.0 (59)	0.3313
Olanzapine	22.3 (344)	23.2 (301)	17.5 (43)	0.0485
Quetiapine	13.6 (210)	13.0 (169)	16.7 (41)	0.1261
Aripiprazole	12.1 (186)	12.1 (157)	11.8 (29)	0.8921
Risperidone	5.2 (80)	5.2 (68)	4.9 (12)	0.8149
Levomepromazine	3.4 (52)	3.3 (43)	3.7 (9)	0.7828
Any LAI	3.9 (60)	3.6 (47)	5.3 (13)	0.2158

IQR, interquartile range; LAI, long-acting injectable antipsychotic.

a. *P*-value: chi-squared test for categorical variables and Kruskal–Wallis test for continuous variables, comparing those who re-started clozapine with those who did not.

b. *n* = 1544 initiated some antipsychotic drug during the first year after clozapine discontinuation (*n* = 1298 among those who did not re-start clozapine and *n* = 379 among those who re-started clozapine during follow-up); in these rows, percentages (*n*) of the types of antipsychotics used by these patient (sub)categories are given.

Of the study cohort, 69% (*n* = 1544) initiated some antipsychotic within 1 year after clozapine discontinuation, whereas 706 did not use antipsychotics in out-patient care settings within that year after clozapine discontinuation. Among those who initiated some antipsychotic during that first year after discontinuation of clozapine use, most commonly they changed to antipsychotic polypharmacy (in 27% of cases, *n* = 409, Table 1; this does not include those receiving clozapine as we had excluded persons who reinitiated clozapine in the first year after clozapine discontinuation). The most common monotherapy during that first year after discontinuation of clozapine was oral olanzapine (22%, *n* = 344), followed by quetiapine (14%, *n* = 210) and oral aripiprazole (12%, *n* = 186). LAI was chosen for 4% (*n* = 60) of persons who discontinued clozapine. The types of antipsychotics that were tried first after clozapine discontinuation were similar in both groups (those who did and those who did not re-start clozapine later during follow-up, Table 1). Reinitiation of clozapine at least 1 year after clozapine discontinuation was observed in 17% (*n* = 379) of patients. A diagnosis of neutropenia or agranulocytosis before clozapine discontinuation was recorded in 85 persons and only one of these re-started clozapine. Similarly, of the 37 participants who had suffered from ileus within the 3 months preceding clozapine cessation, none re-started clozapine. A diagnosis of myocarditis was not recorded in any of the study participants (*n* = 2250).

**Fig. 1:**
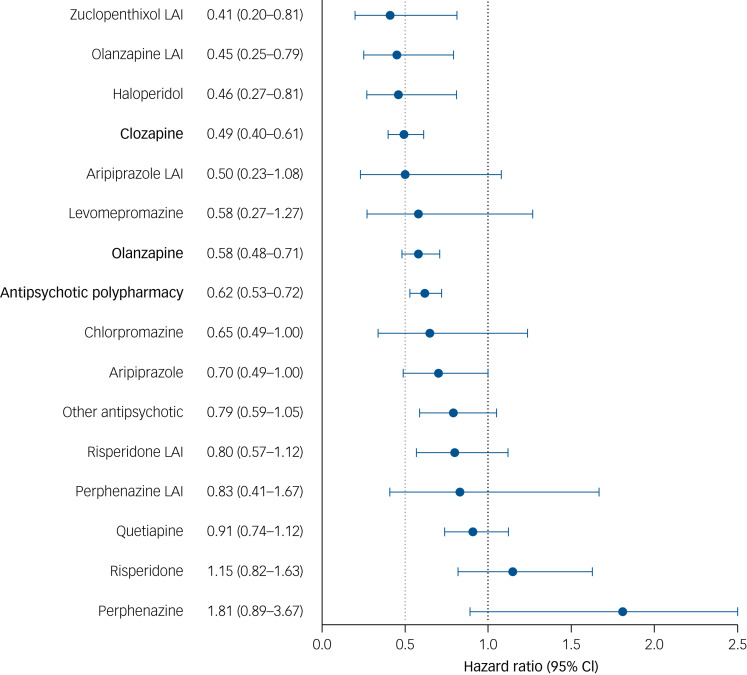
Risk of psychiatric ward readmission for use of antipsychotics compared with non-use of antipsychotics after clozapine discontinuation, within-individual model.

Median follow-up time was 5.4 (interquartile range, 1.4–10.5) years and 50% of the entire study population (*n* = 1122) met the criterion of psychiatric ward readmission during follow-up. The incidence rate of psychiatric ward readmission was 5.3 (95% CI 5.2–5.3) per 10 person-years during antipsychotic use, compared with 6.8 (95% CI 6.6–6.9) per 10 person-years during non-use (aHR 0.66; 95% CI 0.57–0.75; *P* < 0.0001). Of the types of compounds, clozapine (aHR 0.49; 95% CI 0.40–0.61; *P* < 0.0001) and oral olanzapine (aHR 0.58; 95% CI 0.48–0.71; *P* < 0.0001) were significantly associated with the lowest risks of psychiatric ward readmission compared with non-use (Fig. 1). Polypharmacy was also associated with a lower likelihood of psychiatric ward readmission compared with no antipsychotic use (aHR 0.62; 95% CI 0.53–0.72; *P* < 0.0001). When splitting the antipsychotic polypharmacy category into those including and those excluding clozapine, the results did not change (aHR 0.62; 95% CI 0.51–0.75 when including clozapine and aHR 0.63; 95% CI 0.54–0.74 without clozapine).

In bold are depicted the agents that are significant after Bonferroni correction (*P* < 0.003125). Hazard ratios are adjusted for the sequential order of treatments, concomitant use of other psychotropic drugs and time since cohort entry (i.e. the moment of clozapine discontinuation). Antipsychotic polypharmacy refers to the use of two or more antipsychotics concomitantly. LAI, long-acting injectable antipsychotic.

Olanzapine and antipsychotic polypharmacy associations remained significant in the planned sensitivity analysis that restricted inclusion to those who did not re-start clozapine (Supplementary Fig. 2), whereas clozapine and olanzapine remained significant in between-individual sensitivity analyses (Supplementary Fig. 3). Of the atypical antipsychotics, risperidone, quetiapine and aripiprazole, despite having less observations than other atypicals, seem to perform relatively poorly on the outcome of psychiatric ward readmission in clozapine discontinuers (Fig. 1). The numbers of persons, events and incidence rates are provided in Supplementary Table 2.

**Fig. 2:**
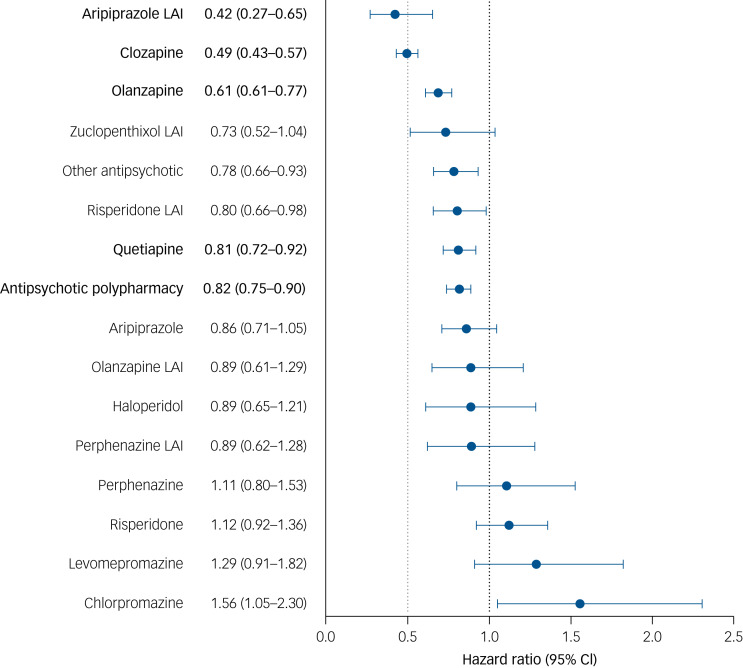
Risk of treatment failure for use of antipsychotics compared with non-use of antipsychotics after clozapine discontinuation, within-individual model.

Of the study cohort, 60% (*n* = 1347) met the criteria for treatment failure. The incidence rate per 10 person-years for treatment failure was 18.1 (95% CI 18.0–18.3) during antipsychotic monotherapy and 21.0 (95% CI 20.8–21.3) during non-use (aHR 0.78; 95% CI 0.72–0.84; *P* < 0.0001) for any antipsychotic compared with non-use. Risk of treatment failure was lowest for aripiprazole LAI (aHR 0.42; 95% CI 0.27–0.65; *P* < 0.0001). Of the types of compounds also associated with lower risk of psychiatric ward readmission than non-use, clozapine (aHR 0.49; 95% CI 0.43–0.57; *P* < 0.0001) and oral olanzapine (aHR 0.69; 95% CI 0.61–0.77; *P* < 0.0001) ranked best in treatment failure analyses (Fig. 2). For this outcome, aripiprazole LAI, olanzapine and clozapine were the only compounds to remain significant after multiple comparisons correction and be confirmed in the planned sensitivity analyses (Supplementary Figs 4 and 5).

In bold are depicted the agents that are significant after Bonferroni correction (*P* < 0.003125). Hazard ratios are adjusted for the sequential order of treatments, concomitant use of other psychotropic drugs and time since cohort entry (i.e. the moment of clozapine discontinuation). Antipsychotic polypharmacy refers to the use of two or more antipsychotics concomitantly. LAI, long-acting injectable antipsychotic.

**Fig. 3:**
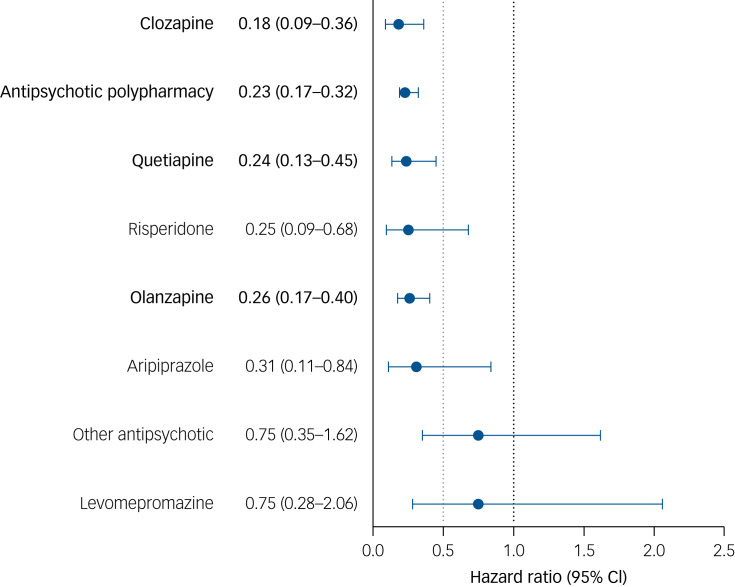
Risk of all-cause mortality for use of antipsychotics compared with non-use of antipsychotics after clozapine discontinuation, between-individual model.

During follow-up, 278 persons died (21.9% unnatural and 78.1% natural/undetermined causes). The incidence rate of death was 0.15 (95% CI 0.14–0.16) per 10 person-years during antipsychotic use and 0.77 (95% CI 0.73–0.81) per 10 person-years during non-use (aHR 0.26; 95% CI 0.20–0.34; *P* < 0.0001), with clozapine (aHR 0.18; 95% CI 0.09–0.36; *P* < 0.0001), antipsychotic polypharmacy (aHR 0.23; 95% CI 0.17–0.32; *P* < 0.0001), quetiapine (aHR 0.24; 95% CI 0.13–0.45; *P* < 0.0001) and olanzapine (aHR 0.26; 95% CI 0.17–0.40; *P* < 0.0001) significantly associated with lower risk of mortality compared with non-use (Fig. 3).

In bold are depicted the agents that are significant after Bonferroni correction (*P* < 0.003125). Hazard ratios are adjusted for gender, age at clozapine discontinuation, number of previous ward admissions owing to psychosis and time since first schizophrenia diagnosis, concomitant use of other medication and comorbidities. Antipsychotic polypharmacy refers to the use of two or more antipsychotics concomitantly.

The two-drug combination results of clozapine, olanzapine, quetiapine, risperidone and aripiprazole are presented in Supplementary Fig. 6 and Table 4. When considering both psychiatric ward readmission and treatment failure, the best combinations were with either clozapine, olanzapine or quetiapine. For psychiatric ward readmission, clozapine with quetiapine performed best (aHR 0.35; 95% CI 0.23–0.53; *P* < 0.0001) but for treatment failure, clozapine with aripiprazole (aHR 0.58; 95% CI 0.47–0.72; *P* < 0.0001) and clozapine with olanzapine (aHR 0.58; 95% CI 0.43–0.78; *P* = 0.0004) carried the lowest risks.

## Discussion

To our knowledge, this is the first study to investigate the comparative effectiveness of a broad range of antipsychotics in patients who, for any reason, terminate clozapine treatment. Our results from a large nationwide cohort, including sensitivity analyses, show that clozapine and oral olanzapine are consistently associated with the best outcomes (i.e. lower risks of psychiatric ward readmission, treatment failure and mortality) compared with no antipsychotic use and other antipsychotics. Contrary to our hypothesis, we thus find evidence that in patients formerly taking clozapine, this compound should be reconsidered.

In all analyses, clozapine was either the best-performing or among the top best-performing antipsychotics, both in monotherapy and two-drug combinations (Supplementary Fig. 6). Several investigators have suggested that long-term administration of antipsychotics induces tolerance to efficacy, especially for clozapine.^17–19^ This could be a reason for physicians to not re-start clozapine therapy after clozapine discontinuation, which was our hypothesis before starting the analyses. Our findings do no support such a notion, as evidenced by the lower risk of psychiatric ward admission, treatment failure and mortality in those who re-start clozapine compared with non-use. Besides concerns about reduced efficacy over time in those who reinitiate clozapine, physicians may worry about the recurrence of serious adverse drug reactions. Our data confirm this suspicion for those patients diagnosed with agranulocytosis/neutropenia under clozapine therapy: only 1 out of 85 patients diagnosed with agranulocytosis/neutropenia re-started clozapine therapy. Importantly, the majority of agranulocytosis/neutropenia cases ascribed to clozapine use occur in the first months of clozapine treatment. Because our study population consists of only those on clozapine for at least a year, most of these 85 are likely to have been neutropenia cases resulting from additional underlying medical conditions (e.g. autoimmune disorders and infections) and comedication (e.g. chemotherapy). On a general note, neutropenia is a more common reason for clozapine discontinuation than agranulocytosis, and about 50% of patients with previous neutropenia tolerate clozapine rechallenge.^26^ Whenever clozapine seems the only viable antipsychotic treatment option in patients with schizophrenia, adding lithium may increase neutrophil counts, but recently authors have cautioned against lithium discontinuation in such patients.^27^ If clinicians are concerned about past or future serious adverse drug reactions in a specific patient previously on clozapine, several good treatment options other than clozapine remain, based on our data. Oral olanzapine was the second-best-performing antipsychotic in our analyses as this compound was always among the four best-performing drugs in both monotherapy and antipsychotic polypharmacy. Such olanzapine findings are in line with a recent meta-analysis showing superior efficacy at the symptom level of olanzapine in TRS.^9^ Another meta-analysis also found that olanzapine was effective in patients with TRS, albeit less effective than clozapine, supporting our findings of better effectiveness of clozapine over olanzapine in this study population.^28^ Aripiprazole (particularly as an LAI, both in monotherapy and antipsychotic polypharmacy) also performed well on most outcome measures. Possibly, adherence is better in persons taking aripiprazole LAI, explaining why this formulation outperforms the oral one. A potential caveat with the aripiprazole LAI analysis are the relatively low numbers of persons and person-years (Supplementary Tables 2 and 3). Because clozapine performs so well in patients who previously used this compound, a final clinical implication of our findings concerns the importance of prevention of clozapine termination in those who did not encounter serious adverse events. To prevent clozapine discontinuation in such patients, conveying a low threshold for asking questions to the prescriber, providing adequate and intelligible information on clozapine, and appropriate dosing (‘start low, go slow’) all add to translating the outcome of this study into clinical practice.

In our study population of clozapine discontinuers, we found no cases of myocarditis. In several countries, including Australia, incidences of clozapine-emergent myocarditis have been reported to be as high as 3%, with average time to event at around 15 days after clozapine initiation. Because, for our study population, we required clozapine use of at least 1 year before discontinuation, it is likely that, such cases of myocarditis were filtered out in our study. In addition, differences in registration/reporting of adverse events and in (genetic) susceptibility to myocarditis may explain disparate clozapine-emergent myocarditis figures across the globe.

In line with recent studies in British and Finnish patients with schizophrenia showing good survival rates for antipsychotic combination therapy, antipsychotic polypharmacy performed well in the current primary and secondary analyses (with slightly weaker results in sensitivity analyses).^14,29^ In particular, the combinations of clozapine with quetiapine, clozapine with olanzapine, clozapine with aripiprazole and quetiapine with risperidone seem promising treatment options based on our data. The current study population consisted of relatively severely affected patients who possibly require treatment with multiple antipsychotics for optimal occupation of the D2 and 5HT2a receptors. Our results show that antipsychotic polytherapy combinations of both clozapine and olanzapine with quetiapine are effective in preventing ward readmission. In a recent paper, Tiihonen *et al* suggested that poor treatment adherence could also be a reason for the positive outcomes of antipsychotic polypharmacy in real-world patients.^14^ Possibly, patients with two antipsychotic prescriptions could decide to use one of these despite poor general pharmacotherapeutic adherence.

The incidence rate of death during antipsychotic use in the current study was similar to a recent study from Taipale *et al* (0.15 per 10 person-years in this study *v.* 0.13 per 10 person-years in that previous study).^30^ The incidence rate of death during non-use in the current study was higher compared with that previous study (0.77 *v.* 0.22), which is likely explained by increased severity of illness in the current study population.^30^ On a general note, mortality in patients with schizophrenia, most of whom are treated with antipsychotics, is two to three times higher than in the general population;^30^ mortality decreases by 56% in patients with schizophrenia on antipsychotics compared with those patients not on antipsychotics.^30^

Strengths of this observational, national registry study include the high statistical power and relative data completeness (with regard to both dependent and independent variables), as RCTs often recruit only a fraction of the study population we assembled and may suffer from high attrition. Moreover, we applied stringent inclusion criteria to our study population, such as ensuring that temporary interruptions of clozapine use would not count as clozapine discontinuation. Nonetheless, a pitfall in observational studies is the occurrence of selection bias. For the current analyses we did not have information about reasons for clozapine cessation at our disposal. Reasons for both clozapine cessation and treatment failure may range from insidious and acute adverse drug reactions to lack of consent to blood monitoring. To minimise potential selection bias, within-individual analyses were applied to our primary research question. These analyses adjust for patient-related characteristics that may affect drug efficacy and tolerability, such as age, gender, comorbidities and time of illness onset. Moreover, the PRE2DUP method was used to model drug use. This method describes actual drug use and has been shown to be reliable.^31^ Based on the current data, we indeed cannot determine whether in those who stopped clozapine because of safety concerns, either clozapine reinstatement or a prescription of a different antipsychotic is preferred. Furthermore, although observational studies do not have the benefit of randomization to reduce the risk of confounding bias in between-individual analyses, we here minimised this caveat through correction for several confounders, such as age, gender, concomitant use of other drugs and the number of previous ward admissions owing to psychosis (Supplementary Table 1). A final limitation is the relative paucity of persons using the following formulations, hampering definite conclusions about their effectiveness in those who discontinue clozapine: oral chlorpromazine, levomepromazine, perphenazine, haloperidol (showing a fairly low hazard ratio for ward readmission, albeit nonsignificant; Fig. 1), zuclopenthixol (showing a fairly low hazard ratio for ward readmission, albeit nonsignificant; Fig. 1) and LAIs (Supplementary Tables 2 and 3). Conversely, given the high number of observations for olanzapine, statistical power to detect positive results for this agent is more substantial than for several other agents, e.g. aripiprazole LAI (with only 30 persons). Of note, amisulpride is not registered in Finland and therefore data on this compound were unavailable for the current analyses.

In conclusion, our results indicate that therapy with antipsychotics is preferred over no use of antipsychotics in patients who discontinue clozapine, both in terms of efficacy and safety. Among those not using antipsychotics after clozapine cessation, we observed a ≥4-fold increased mortality rate compared with those using any antipsychotic. Re-starting clozapine therapy in patients who discontinue clozapine is the most effective and safe treatment option for most patients, with the exception of those discontinuing clozapine therapy because of serious adverse events, for whom clinicians should consider prescribing olanzapine.

## Supplementary material

For supplementary material accompanying this paper visit https://doi.org/10.1192/bjp.2019.267. click here to view supplementary material
