# Supplementary material for: In the aftermath of clozapine discontinuation: comparative effectiveness and safety of antipsychotics in patients with schizophrenia who discontinue clozapine
Source: Br J Psychiatry. 2020 Sep;217(3):498–505. doi: 10.1192/bjp.2019.267 (PMC7511905; doi:10.1192/bjp.2019.267)
Supplement: Supplementary file 1 [file S0007125019002678sup001.docx]

**Supplemental Material**

**Supplementary Figure 1.** Flow chart of exclusions for the study.


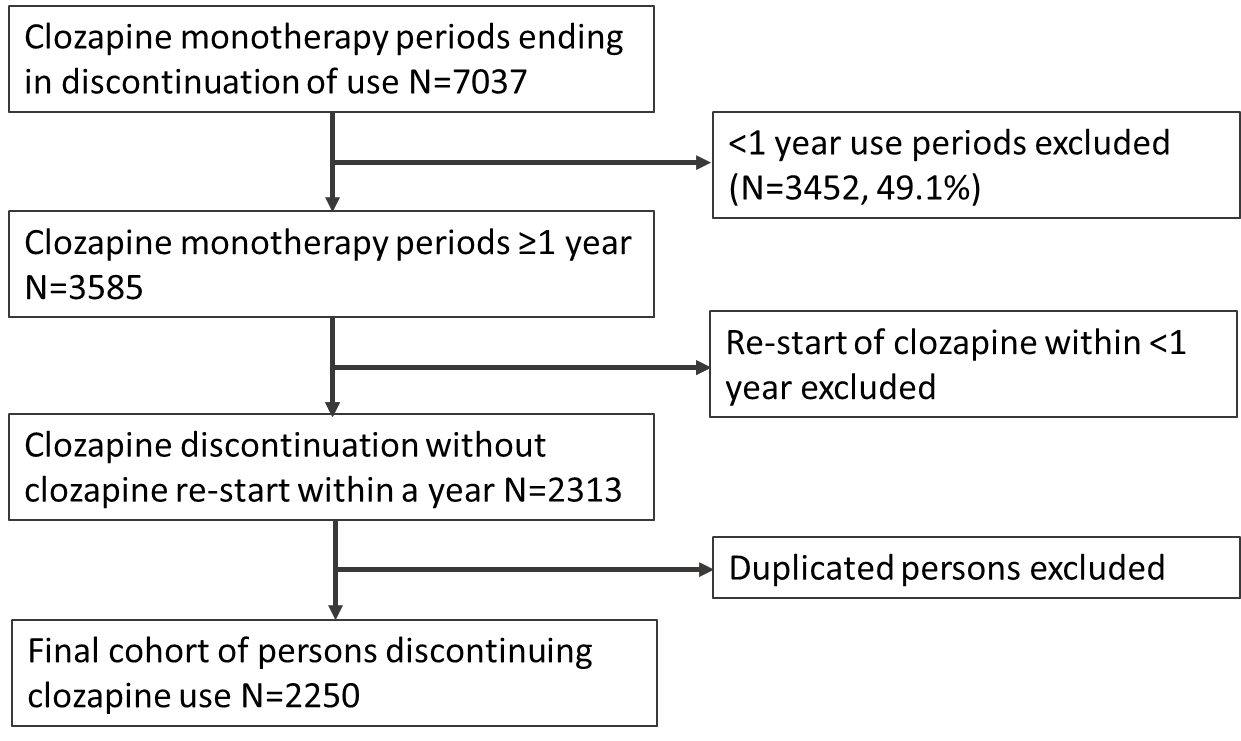


**Supplementary Figure** 2. Risk of *psychiatric hospitalization* for use of antipsychotics compared with non-use of antipsychotics after clozapine discontinuation among those who did not re-start clozapine use during the follow-up, within-individuals model. In bold are depicted the agents that are significant after Bonferroni correction (*P*<0.003125). HR = hazard ratio, adjusted for the sequential order of treatments, concomitant use of other psychotropic drugs, and time since cohort entry (i.e. the moment of clozapine discontinuation). Antipsychotic (AP) polypharmacy refers to the use of ≥2 APs concomitantly.


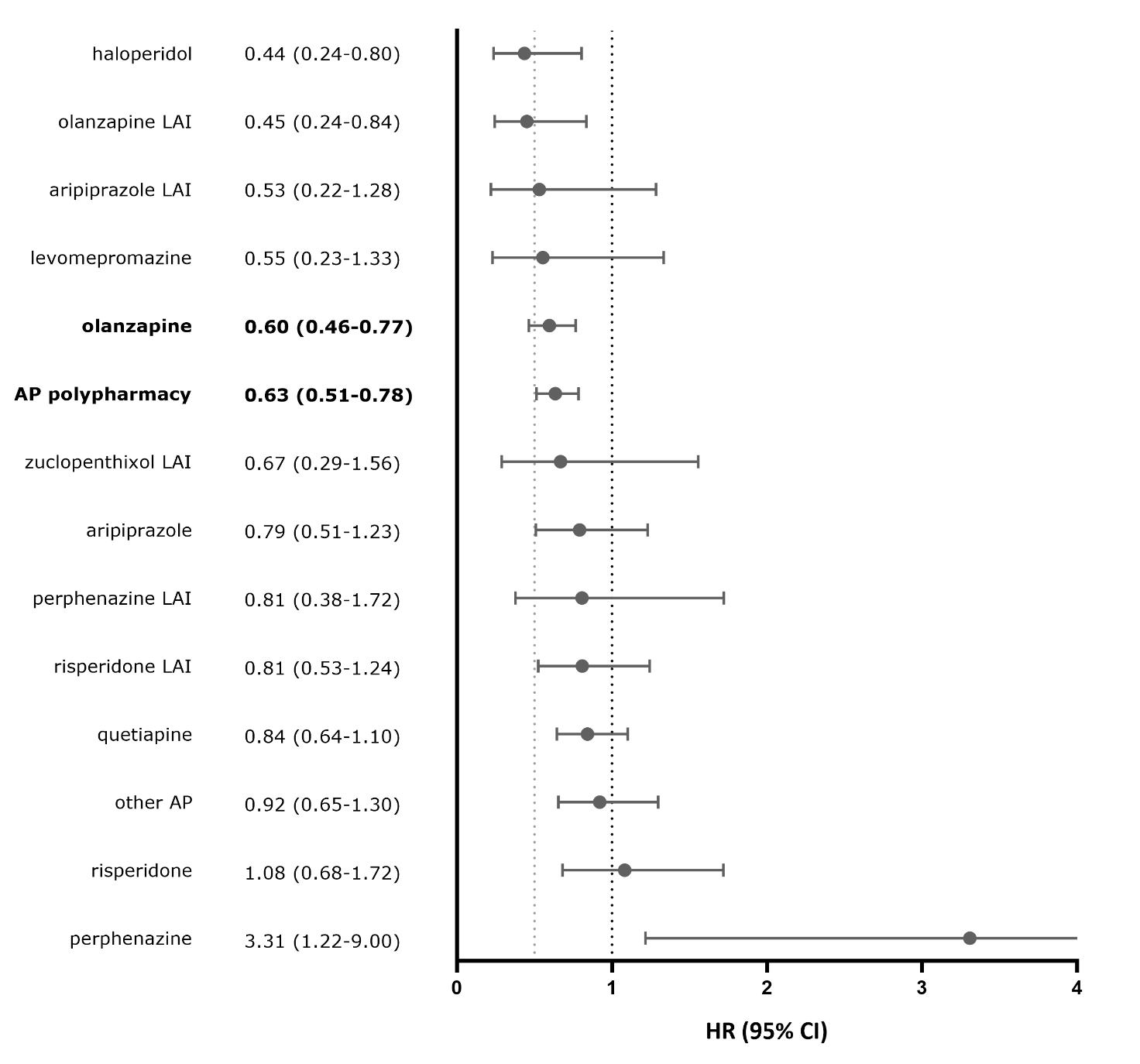


**Supplementary Figure** **3**. Risk of *psychiatric hospitalization* for use of antipsychotics compared with non-use of antipsychotics after clozapine discontinuation, between-individuals model. In bold are depicted the agents that are significant after Bonferroni correction (*P*<0.003125). HR = hazard ratio, adjusted for sex, age at clozapine discontinuation, number of previous hospitalizations due to psychosis, time since first schizophrenia diagnosis, and concomitant use of other medication and comorbidities. Antipsychotic (AP) polypharmacy refers to the use of ≥2 APs concomitantly.


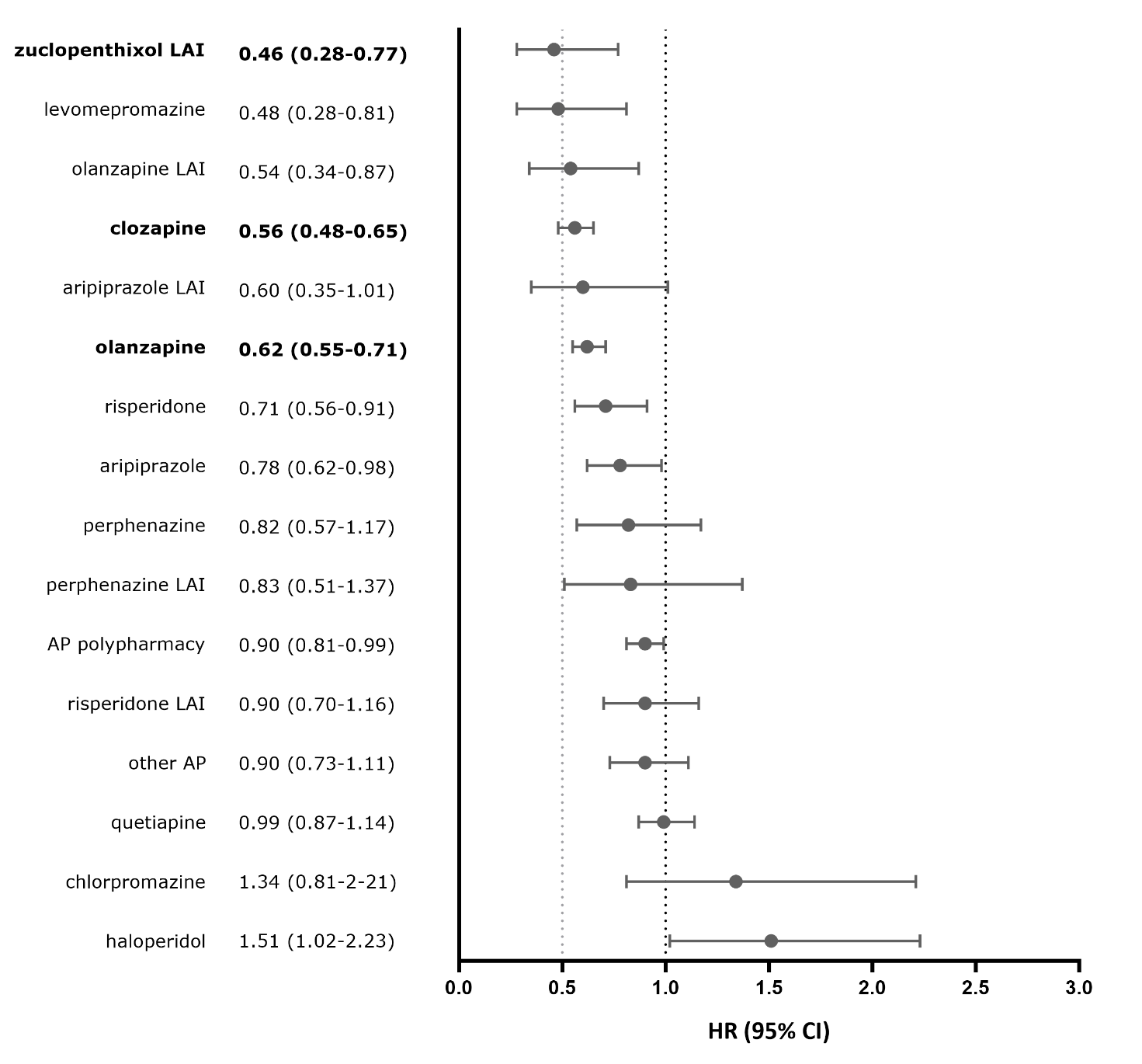


**Supplementary Figure** **4**. Risk of *treatment failure* for use of antipsychotics compared with non-use of antipsychotics after clozapine discontinuation among those who did not re-start clozapine use during the follow-up, within-individual model. In bold are depicted the agents that are significant after Bonferroni correction (*P*<0.003125). HR = hazard ratio, adjusted for the sequential order of treatments, concomitant use of other psychotropic drugs, time since cohort entry (i.e. the moment of clozapine discontinuation). Antipsychotic (AP) polypharmacy refers to the use of ≥2 APs concomitantly.


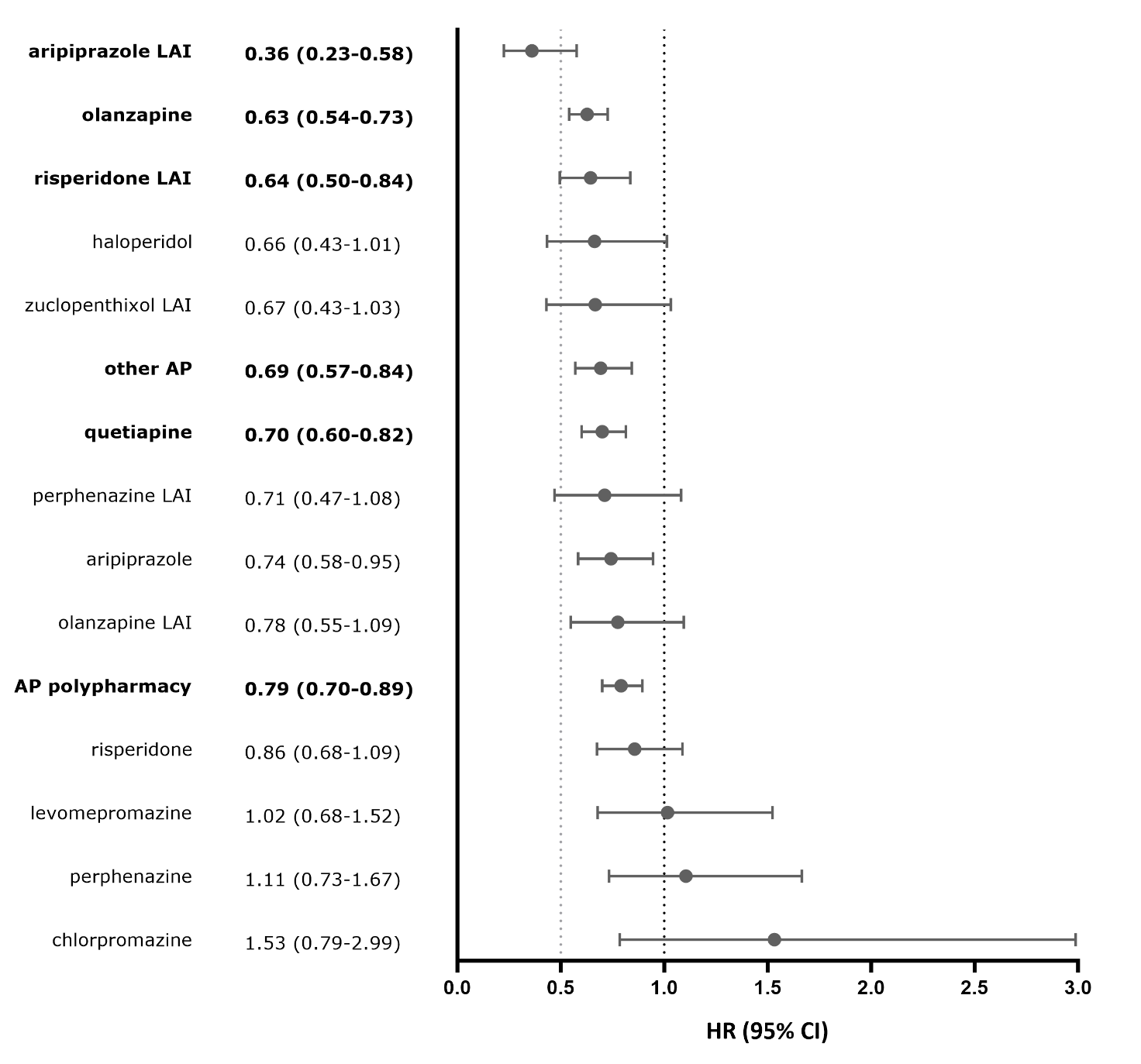


**Supplementary Figure** **5**. Risk of *treatment failure* for use of antipsychotics compared with non-use of antipsychotics after clozapine discontinuation, between-individuals model. In bold are depicted the agents that are significant after Bonferroni correction (*P*<0.003125). HR = hazard ratio, adjusted for sex, age at clozapine discontinuation, number of previous hospitalizations due to psychosis, time since first schizophrenia diagnosis, concomitant use of other medication and comorbidities. Antipsychotic (AP) polypharmacy refers to the use of ≥2 APs concomitantly.


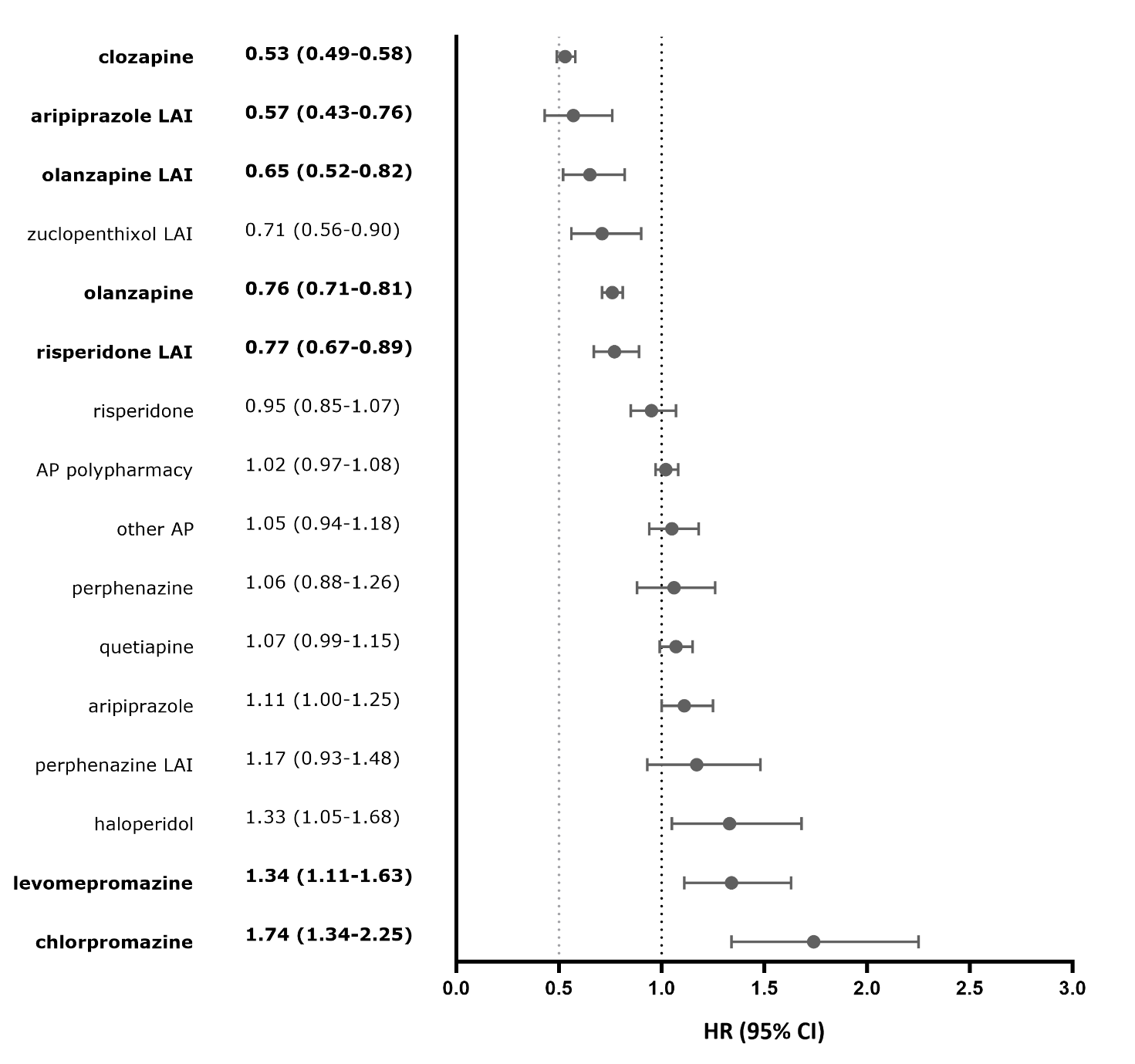


**Supplementary figure 6.** Risk of treatment failure and psychiatric rehospitalization associated with the five most commonly used oral antipsychotics as *two-drug combinations*, compared with no antipsychotic use. HR = hazard ratio, adjusted for the sequential order of treatments, concomitant use of other psychotropic drugs, and time since cohort entry (i.e. the moment of clozapine discontinuation).


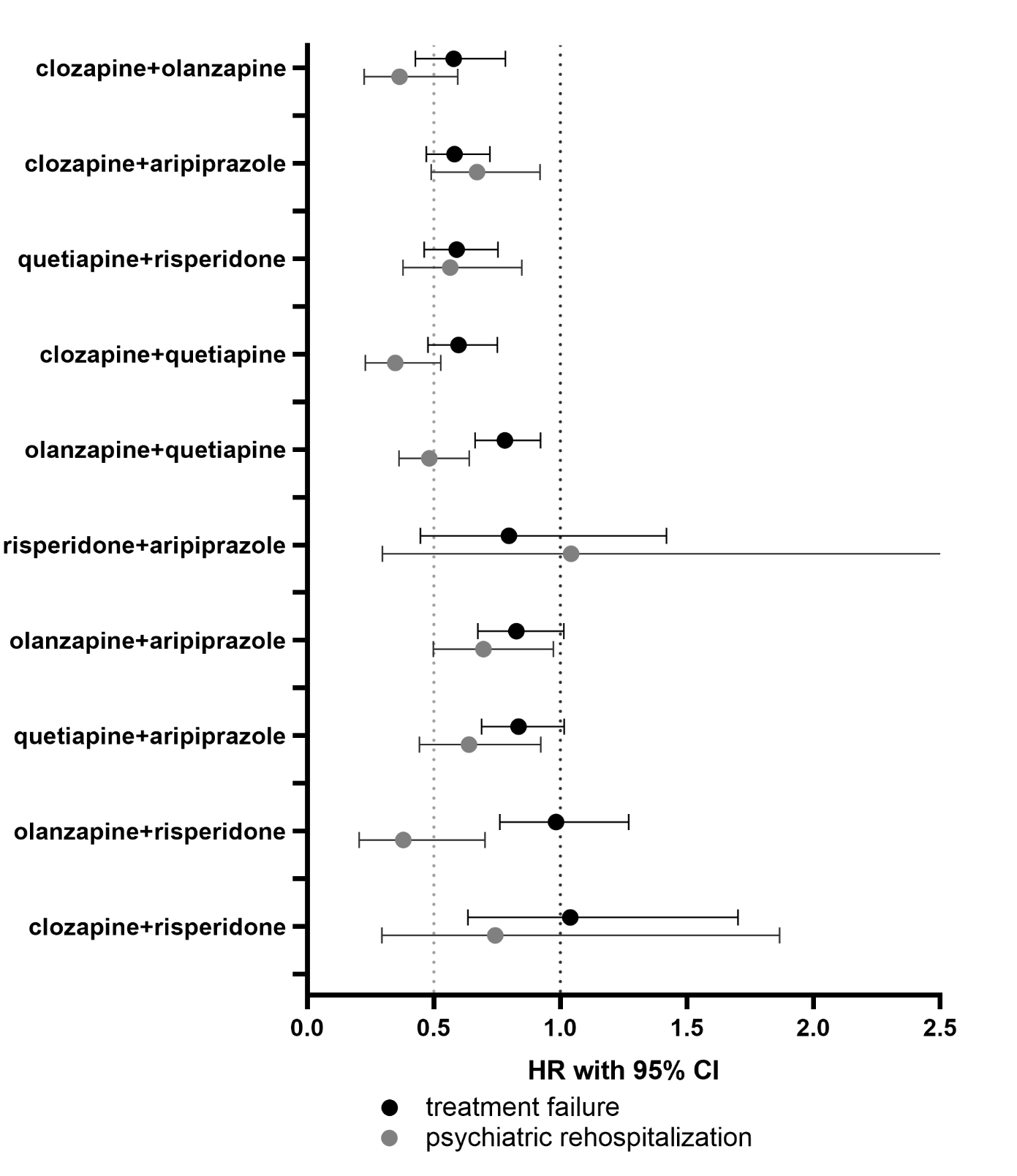


| **Supplementary Table 1. Covariate definitions for between-individuals analyses.** | | |
| --- | --- | --- |
| **Covariate** | **Definition** | **Register** |
| Order of treatments | \| Order of treatment continuously updated in the models, categorized as no treatment, 1st, 2nd, 3rd, >3rd \| \| --- \| | PR |
| Concomitant use of other psychotropic drugs | Antidepressants (N06A), mood stabilizers (valproate N03AG01, carbamazepine N03AF01, lamotrigine N03AX09), lithium (N05AN01), benzodiazepines and related drugs (N05BA, N05CD, N05CF) continuously updated in the models | PR |
| Time since cohort entry | Cohort entry refers to date of clozapine discontinuation, continuously updated in the models | PR |
| **Other medication use**: defined time-dependently during the follow-up (current use vs. no use currently) | | |
| Lipid-modifying agents | C10A, C10B | PR |
| Opioid analgesics | N02A | PR |
| Non-opioid analgesics | N02BE01, M01A | PR |
| Anti-Parkinson drugs | N04 | PR |
| Prior use of LAI | Prior use, yes or no | PR |
| **Comorbidities**: continuously updated variables with status ”no” until the first instance of diagnoses occurred and ”yes” thereafter. | | |
| Substance abuse | F10-16 and/or F18-19 (Mental and behavioral disorders due to psychoactive substance use)  K70 (Alcoholic liver disease)  K86.0 (Alcohol-induced chronic pancreatitis)  “reason for admission” codes referring to substance abuse (33\|71\|72\|73\|74\|75)  ATC-codes:  N07BB (Drugs used in alcohol dependence)  N07BC (Drugs used in opioid dependence) | HDR, PR |
| Cardiovascular disease | I00-I99 | HDR |
| Previous suicide attempt | X60-84, Y10-34, Z728, Z915 | HDR |
| Diabetes | E10-E14, or antidiabetic use A10 | HDR, PR |
| Asthma/COPD | J42-44 | HDR |
| Previous cancer | C01-C99 | HDR |
| Renal disease | N10-N19 | HDR |
| Age | At clozapine discontinuation; ≤35, 36-55, >55 |  |
| Gender |  |  |
| The number of previous hospitalizations due to psychosis | Main diagnoses of F20-29;  categorized as ≤1, 2-3, >3 | HDR |
| Time since first schizophrenia diagnoses | In years, categorized as ≤1, >1-5, >5 |  |
| HDR: Hospital Discharge Register, PR: Prescription register | | |

| **Supplementary Table 2. The number of users, events and person-years, and incidence rate of psychiatric rehospitalization among the cohort of person who discontinued clozapine use. Whenever ‘LAI’ is not mentioned, the oral formulation is meant. AP: antipsychotic.** | | | | |
| --- | --- | --- | --- | --- |
|  | Users | Events | Person-years | Incidence rate per 10 person-years  (95% CI) |
| non-use | 967 | 791 | 1166.08 | 6.8 (6.6-6.9) |
| chlorpromazine | 29 | 16 | 8.89 | 18.0 (15.2-20.8) |
| levomepromazine | 77 | 14 | 36.51 | 3.8 (3.2-4.5) |
| perphenazine | 61 | 31 | 50.15 | 6.2 (5.5-6.9) |
| perphenazine LAI | 25 | 16 | 27.35 | 5.9 (4.9-6.8) |
| haloperidol | 48 | 26 | 29.92 | 8.7 (7.6-9.7) |
| zuclopenthixol LAI | 39 | 15 | 42.23 | 3.6 (3.0-4.1) |
| clozapine | 560 | 265 | 896.57 | 3.0 (2.8-3.1) |
| olanzapine | 435 | 383 | 1003.62 | 3.8 (3.7-3.9) |
| olanzapine LAI | 42 | 18 | 44.97 | 4.0 (3.4-4.6) |
| quetiapine | 311 | 349 | 490.6 | 7.1 (6.9-7.3) |
| risperidone | 148 | 74 | 199.45 | 3.7 (3.4-4.0) |
| risperidone LAI | 86 | 69 | 124.73 | 5.5 (5.1-5.9) |
| aripiprazole | 163 | 82 | 139.18 | 5.9 (5.5-6.3) |
| aripiprazole LAI | 31 | 14 | 27.16 | 5.2 (4.3-6.0) |
| other AP | 159 | 104 | 152.87 | 6.8 (6.4-7.2) |
| AP polypharmacy | 1062 | 2292 | 3888.47 | 5.9 (5.8-6.0) |

| **Supplementary Table 3. The number of users, events and person-years, and incidence rate of psychiatric rehospitalization for persons who did not re-start clozapine use during the follow-up and for between individual analyses. AP: antipsychotic.** | | | | | | | | |
| --- | --- | --- | --- | --- | --- | --- | --- | --- |
|  | Those who did not re-start clozapine use | | | | Between individual comparison | | | |
|  | Users | Events | Person-years | Incidence rate per 10 person-years  (95% CI) | Users | Events | Person-years | Incidence rate per 10 person-years  (95% CI) |
| non-use | 604 | 445 | 779.4 | 5.7 (5.5-5.9) | 1434 | 791 | 1835.87 | 4.3 (4.2-4.4) |
| chlorpromazine | 16 | 1 | 4.52 | 2.2 (0.8-3.6) | 34 | 16 | 16.31 | 9.8 (8.3-11.3) |
| levomepromazine | 56 | 11 | 32.81 | 3.4 (2.7-4.0) | 98 | 14 | 57.54 | 2.4 (2.0-2.8) |
| perphenazine | 40 | 25 | 38.94 | 6.4 (5.6-7.2) | 80 | 31 | 65.17 | 4.8 (4.2-5.3) |
| perphenazine LAI | 17 | 14 | 25.19 | 5.6 (4.6-6.5) | 27 | 16 | 29.95 | 5.3 (4.5-6.2) |
| haloperidol | 38 | 24 | 28.2 | 8.5 (7.4-9.6) | 57 | 26 | 35.31 | 7.4 (6.5-8.3) |
| zuclopenthixol LAI | 28 | 11 | 29 | 3.8 (3.1-4.5) | 43 | 15 | 45.64 | 3.3 (2.8-3.8) |
| clozapine | 0 | 0 | 0 | NA | 908 | 265 | 1005.2 | 2.6 (2.5-2.7) |
| olanzapine | 331 | 310 | 850.64 | 3.6 (3.5-3.8) | 577 | 383 | 1578.99 | 2.4 (2.3-2.5) |
| olanzapine LAI | 35 | 16 | 41.69 | 3.8 (3.2-4.4) | 45 | 18 | 47.01 | 3.8 (3.3-4.4) |
| quetiapine | 219 | 257 | 411.67 | 6.2 (6.0-6.5) | 416 | 349 | 667.99 | 5.2 (5.1-5.4) |
| risperidone | 110 | 46 | 169.97 | 2.7 (2.5-3.0) | 174 | 74 | 219.61 | 3.4 (3.1-3.6) |
| risperidone LAI | 56 | 46 | 95.75 | 4.8 (4.4-5.2) | 90 | 69 | 126.07 | 5.5 (5.1-5.9) |
| aripiprazole | 122 | 67 | 116.3 | 5.8 (5.3-6.2) | 269 | 82 | 279.27 | 2.9 (2.7-3.1) |
| aripiprazole LAI | 30 | 12 | 26.59 | 4.5 (3.7-5.3) | 38 | 14 | 30.33 | 4.6 (3.9-5.4) |
| other AP | 123 | 88 | 134.03 | 6.6 (6.1-7.0) | 192 | 104 | 194.87 | 5.3 (5.0-5.7) |
| AP polypharmacy | 655 | 1375 | 2594.05 | 5.3 (5.2-5.4) | 1534 | 2292 | 4566.94 | 5.0 (5.0-5.1) |

| **Supplementary Table 4.** Two-drug combinations of five most commonly used oral antipsychotics and associated risk of psychiatric rehospitalization and treatment failure compared with no antipsychotic use. | | | | | | | | |
| --- | --- | --- | --- | --- | --- | --- | --- | --- |
|  | | | Psychiatric rehospitalization | | | Treatment failure | | |
| drugs | users | person-years | events | incidence rate per 10 person-years (95% CI) | aHR (95% CI) | events | incidence rate per 10 person-years (95% CI) | aHR (95% CI) |
| clozapine, olanzapine | 168 | 44.53 | 28 | 6.3 (5.6-7.0) | 0.37 (0.23-0.59) | 221 | 49.6 (47.6-51.7) | 0.58 (0.43-0.78) |
| clozapine, quetiapine | 146 | 133.89 | 40 | 3.0 (2.7-3.3) | 0.35 (0.23-0.53) | 283 | 21.1 (20.4-21.9) | 0.60 (0.48-0.75) |
| clozapine, risperidone | 50 | 50.16 | 9 | 1.8 (1.4-2.2) | 0.74 (0.30-1.98) | 74 | 14.8 (13.7-15.8) | 1.04 (0.64-1.70) |
| clozapine, aripiprazole | 133 | 194.33 | 102 | 5.2 (4.9-5.6) | 0.67 (0.49-0.92) | 305 | 15.7 (15.1-16.3) | 0.58 (0.47-0.72) |
| olanzapine, quetiapine | 169 | 309.88 | 120 | 3.9 (3.7-4.1) | 0.48 (0.36-0.64) | 513 | 16.6 (16.1-17.0) | 0.78 (0.66-0.92) |
| olanzapine, risperidone | 81 | 77.95 | 20 | 2.6 (2.2-2.9) | 0.38 (0.21-0.70) | 157 | 20.1 (19.1-21.1) | 0.98 (0.76-1.27) |
| olanzapine, aripiprazole | 122 | 113.2 | 76 | 6.7 (6.2-7.2) | 0.70 (0.50-0.97) | 263 | 23.2 (22.3-24.1) | 0.83 (0.67-1.01) |
| quetiapine, risperidone | 59 | 87.83 | 70 | 8.0 (7.4-8.6) | 0.57 (0.38-0.85) | 191 | 21.7 (20.8-22.7) | 0.59 (0.46-0.75) |
| quetiapine, aripiprazole | 113 | 131.64 | 60 | 4.6 (4.2-4.9) | 0.64 (0.44-0.92) | 311 | 23.6 (22.8-24.5) | 0.84 (0.69-1.02) |
| risperidone, aripiprazole | 20 | 6.99 | 5 | 7.2 (5.2-9.1) | 1.04 (0.30-3.65) | 34 | 48.6 (43.5-53.8) | 0.80 (0.45-1.42) |
